# Psychological Well-Being and Oral Functional Recovery Following Combined Dental and Facial Reconstruction After Traumatic Jaw Loss: A Prospective Study

**DOI:** 10.7759/cureus.101959

**Published:** 2026-01-21

**Authors:** Hassan Masood, Mamoona Manzoor, Lojain Maqsood, Junaid Israr Ahmed Khan, Samreen Fatima, Saher Sultan, Usman Mahmood, Razwan Ashraf

**Affiliations:** 1 Oral and Maxillofacial Surgery, Al-Muhaidib Dental Group, Jeddah, SAU; 2 Psychiatry, Pakistan Railway Hospital, Rawalpindi, PAK; 3 Psychiatry, Gulab Devi Teaching Hospital, Lahore, PAK; 4 Orthodontics, Lahore Medical and Dental College, Lahore, PAK; 5 Psychiatry, Abu Umaara Medical College, Ali Fatima Hospital, Lahore, PAK; 6 Community and Preventive Dentistry, National University of Medical Sciences, Rawalpindi, PAK; 7 Science of Dental Materials, Lahore Medical and Dental College, Lahore, PAK; 8 Medicine, Aziz Bhatti Shaheed Teaching Hospital, Gujrat, PAK

**Keywords:** masticatory function, maxillofacial trauma, oral rehabilitation, prospective cohort study, psychological outcomes

## Abstract

Background

Traumatic jaw loss results in profound functional impairment and psychological distress, substantially affecting the quality of life of patients. While advances in dental and facial reconstruction have improved anatomical and functional outcomes, prospective evidence linking oral functional recovery with psychological well-being remains limited, particularly in low- and middle-income settings.

Objectives

The objectives of this study are (i) to prospectively evaluate changes in psychological well-being and oral functional recovery in patients following combined dental and facial reconstruction after traumatic jaw loss and (ii) to examine the association between functional outcomes and psychological well-being.

Methods

This prospective observational cohort study was conducted over 12 months at Abu Umaara Medical College, Ali Fatima Hospital in Lahore (Pakistan). Adult patients with traumatic mandibular and/or maxillary defects undergoing combined dental and facial reconstruction were enrolled using non-probability consecutive sampling. Psychological well-being was assessed using the World Health Organization-Five Well-Being Index (WHO-5). Oral functional recovery was evaluated through maximal interincisal mouth opening, masticatory efficiency, speech intelligibility, and patient-reported oral function. Assessments were performed preoperatively and at three and six months postoperatively. Repeated-measures analysis of variance (ANOVA), correlation analysis, and multivariable linear regression were applied, with p<0.05 considered statistically significant.

Results

Of the 132 eligible patients, 120 were enrolled and 112 completed the six-month follow-up. The mean age was 38.6±11.2 years, and 72.5% of the participants were men. Baseline WHO-5 scores indicated poor psychological well-being (34.8±9.6), alongside marked oral functional impairment. Significant improvements were observed at three and six months in WHO-5 scores and all functional parameters (all p<0.001). At six months, psychological well-being showed strong positive correlations with mouth opening (r=0.62), masticatory efficiency (r=0.68), speech intelligibility (r=0.55), and patient-reported oral function (r=0.71). Multivariable regression identified masticatory efficiency and patient-reported oral function as independent predictors of psychological well-being, explaining 58% of variance in WHO-5 scores.

Conclusion

Combined dental and facial reconstruction after traumatic jaw loss leads to significant improvements in oral function and psychological well-being. Functional recovery, particularly masticatory efficiency and perceived oral function, plays a pivotal role in psychological rehabilitation, underscoring the importance of integrated, patient-centered reconstructive care.

## Introduction

Traumatic injuries to the maxillofacial region represent a significant global health burden and are frequently associated with high-impact mechanisms such as road traffic accidents, interpersonal violence, and occupational trauma. Beyond the immediate physical damage, traumatic jaw loss often results in complex functional deficits involving mastication, speech, swallowing, and facial aesthetics, which can profoundly disrupt daily living and social interaction. While surgical reconstruction has traditionally focused on restoring anatomical continuity and facial symmetry, there is growing recognition that the consequences of such injuries extend well beyond physical impairment and encompass substantial psychological morbidity [1,2].

The psychological sequelae of maxillofacial trauma are increasingly documented in the literature, with high reported rates of anxiety, depression, post-traumatic stress disorder, and reduced quality of life following facial injury [1]. Changes in facial appearance, loss of oral function, and altered self-perception can negatively affect self-esteem, interpersonal relationships, and social participation. Walshaw et al. highlighted that psychological distress following oral and maxillofacial trauma is frequently under-recognized in routine surgical follow-up despite clear evidence that these sequelae may persist for months or years after injury [3]. Importantly, the literature also demonstrates considerable heterogeneity in psychological outcomes, influenced by injury severity, functional impairment, comorbidities, and access to rehabilitative care [4,5].

Restoration of oral function plays a pivotal role in post-traumatic recovery. Previous studies have shown that impaired mastication, restricted mouth opening, and compromised speech are strongly associated with poorer health-related quality of life and psychological distress after facial trauma [6]. Dental rehabilitation, particularly when integrated with facial reconstruction, has been shown to improve functional outcomes and patient satisfaction; however, its relationship with psychological recovery remains insufficiently explored [7]. Many existing studies focus primarily on psychological outcomes or physical reconstruction in isolation, with limited prospective data examining the interaction between functional recovery and psychological well-being over time [1,3].

Furthermore, the available literature reveals a lack of standardized prospective assessments combining objective oral functional parameters with validated measures of psychological well-being in patients undergoing reconstruction for traumatic jaw loss [8,9]. This gap is particularly evident in low- and middle-income settings, where trauma burden is high and comprehensive rehabilitative services are often limited. Understanding how improvements in oral function translate into psychological recovery is essential for developing holistic, patient-centered treatment pathways and for identifying patients at risk of suboptimal outcomes. The objective of this prospective study was to evaluate changes in psychological well-being and oral functional recovery over time and to examine the association between functional rehabilitation and psychological outcomes in patients undergoing combined dental and facial reconstruction after traumatic jaw loss.

## Materials and methods

This study was designed as a prospective observational cohort study and was reported in accordance with the Strengthening the Reporting of Observational Studies in Epidemiology (STROBE) guidelines. The research was conducted at Abu Umaara Medical College, Ali Fatima Hospital in Lahore, Pakistan. The study duration was 12 months, commencing in August 2023 and concluding in July 2024.

The study population consisted of adult patients presenting with traumatic partial or complete jaw loss who underwent combined dental and facial reconstructive procedures. A sample size of 120 participants was calculated using OpenEpi software (developed by a team of A.G. Dean, K.M. Sullivan and M.M. Soe; OpenEpi: Open Source Epidemiologic Statistics for Public Health), assuming a medium effect size (Cohen’s d=0.5) for change in psychological well-being scores before and after reconstruction, a confidence level of 95%, power of 80%, and an anticipated attrition rate of 10% [10-12]. Non-probability consecutive sampling was employed, whereby all eligible patients meeting the inclusion criteria during the study period were invited to participate until the required sample size was achieved.

Patients aged 18-65 years of either sex, with traumatic mandibular and/or maxillary defects requiring combined dental rehabilitation (implant-supported prosthesis or removable prosthesis) and facial reconstruction (local, regional, or free flap-based reconstruction), and who were medically stable for surgery, were included. Patients with pre-existing diagnosed psychiatric disorders, cognitive impairment limiting reliable questionnaire responses, pathological jaw defects due to malignancy or osteonecrosis, previous reconstructive surgery for the same defect, or those unwilling to provide informed consent were excluded.

Psychological well-being was assessed using the World Health Organization-Five Well-Being Index (WHO-5), a freely available, non-proprietary instrument that does not require prior permission for research use. The WHO-5 consists of five positively worded items scored on a six-point Likert scale ranging from 0 (at no time) to 5 (all of the time), giving a raw score of 0-25. The raw score was multiplied by four to obtain a percentage score ranging from 0 to 100, with higher scores indicating better psychological well-being. Scores ≥50 were considered indicative of good well-being, while scores <50 suggested reduced well-being and potential risk for depression [13]. Oral functional recovery was evaluated using objective and subjective measures, including maximal interincisal mouth opening measured with a calibrated digital caliper. Masticatory efficiency was assessed using a standardized color-change chewing gum test. Participants chewed the gum for 30 standardized strokes. The gum changes from yellow to red, depending on mastication,was compared to a 1-5 color scale, with 1=poor mastication and 5=excellent mastication [14]. Speech intelligibility was assessed using the Intelligibility in Context Scale (ICS), a freely available, validated tool. Participants self-rated seven items on a five-point Likert scale (1=poor intelligibility, 5=excellent). The mean score was calculated, with higher scores indicating better speech intelligibility [15]. Patient-reported oral function was assessed using a freely available Masticatory Function Questionnaire (MFQ). Participants rated 10 items on a five-point Likert scale (0=never able, 4=always able), with total scores ranging from 0 to 40. Higher scores indicate better oral function [16]. Baseline assessments were performed preoperatively, with follow-up evaluations at three and six months post-reconstruction.

Relevant demographic and clinical variables, including age, sex, type and extent of jaw defect, mechanism of trauma, type of reconstructive technique, prosthetic modality, duration between trauma and reconstruction, smoking status, and presence of comorbidities such as diabetes mellitus, were recorded to identify potential confounders. These variables were accounted for during statistical analysis through stratification and multivariable regression modeling. Missing data were minimized through scheduled follow-up reminders; however, in cases where missing values occurred, multiple imputation techniques were applied if missingness exceeded 5%, assuming data were missing at random.

Data were entered and analyzed using SPSS version 26.0 (IBM Corp, Armonk, NY). Continuous variables were expressed as mean±standard deviation or median with interquartile range based on data distribution assessed by the Shapiro-Wilk test. Categorical variables were presented as frequencies and percentages. Changes in psychological well-being and oral functional outcomes over time were analyzed using repeated-measures analysis of variance (ANOVA) or Friedman test as appropriate. Associations between psychological well-being and oral functional recovery were assessed using Pearson or Spearman correlation coefficients, while multivariable linear regression was used to adjust for confounding variables. A p-value of less than 0.05 was considered statistically significant.

Ethical approval for the study was obtained from the Ethical Review Committee of Abu Umaara Medical College, Ali Fatima Hospital Lahore, Pakistan (Reference No. 214/AUMDC/ERC dated March 21, 2023). Written informed consent was obtained from all participants prior to enrollment, and confidentiality of patient data was maintained throughout the study in accordance with the Declaration of Helsinki.

## Results

A total of 132 eligible patients were approached during the study period; 120 consented and were enrolled, yielding a response rate of 90.9%. During follow-up, eight patients were lost at six months, resulting in a final analytical sample of 112 participants. Missing data constituted 4.6% of total observations and were handled using multiple imputation as predefined in the methodology.

The baseline demographic and clinical characteristics of the study participants are presented in Table 1. The mean age of the cohort was 38.6±11.2 years, with a male predominance (72.5%). Road traffic accidents were the most common mechanism of injury (58.3%), followed by interpersonal violence (26.7%) and occupational trauma (15.0%). Mandibular defects alone were observed in 54.2% of patients, maxillary defects in 25.8%, and combined defects in 20.0%. Free flap reconstruction was performed in 47.5% of cases, while regional and local flaps were used in 32.5% and 20.0%, respectively.

**Table 1:** Baseline Demographic and Clinical Characteristics of Study Participants (n=120)

Variable	Value
Age (years), mean±SD	38.6±11.2
Male sex, n (%)	87 (72.5)
Smoking status, n (%)	42 (35.0)
Diabetes mellitus, n (%)	18 (15.0)
Mechanism of trauma, n (%)	
- Road traffic accident	70 (58.3)
- Interpersonal violence	32 (26.7)
- Occupational injury	18 (15.0)
Type of jaw defect, n (%)	
- Mandible only	65 (54.2)
- Maxilla only	31 (25.8)
- Combined	24 (20.0)
Reconstructive technique, n (%)	
- Free flap	57 (47.5)
- Regional flap	39 (32.5)
- Local flap	24 (20.0)

Baseline psychological well-being and oral functional parameters are summarized in Table 2. Preoperatively, the mean WHO-5 score was 34.8±9.6, indicating poor psychological well-being. Oral functional impairment was substantial, with a mean maximal interincisal opening of 21.4±6.3 mm and low masticatory efficiency scores.

**Table 2:** Baseline Psychological and Oral Functional Parameters (Preoperative) (n=120)

Parameter	Mean ± SD
WHO-5 well-being score	34.8±9.6
Maximal interincisal opening (mm)	21.4±6.3
Masticatory efficiency score	4.1±1.2
Speech intelligibility score	2.6±0.7
Patient-reported oral function score	28.3±6.9

Significant improvements were observed in psychological well-being and oral functional outcomes at three and six months postoperatively. Repeated-measures ANOVA demonstrated a statistically significant increase in WHO-5 scores over time (F=112.4, p<0.001). Similarly, maximal mouth opening, masticatory efficiency, speech intelligibility, and patient-reported oral function showed significant time-dependent improvement (all p<0.001). These changes are detailed in Table 3.

**Table 3:** Changes in Psychological Well-Being and Oral Functional Outcomes Over Time (n=112) Repeated-measures ANOVA was used to assess changes over time. Baseline (preoperative) values in this table are calculated from the analytic sample of patients who completed follow-up (n = 112) and were used for the repeated-measures analysis.

Parameter	Preoperative	3 Months	6 Months	Test Statistic	p-value
WHO-5 score	34.8±9.6	52.7±10.4	63.9±9.1	F=112.4	<0.001
Mouth opening (mm)	21.4±6.3	31.6±5.8	38.9±5.1	F=156.2	<0.001
Masticatory efficiency	4.1±1.2	6.8±1.4	8.2±1.3	F=141.7	<0.001
Speech intelligibility	2.6±0.7	3.7±0.6	4.3±0.5	F=98.5	<0.001
Oral function score	28.3±6.9	39.5±7.2	46.8±6.4	F=124.9	<0.001

Correlation analysis revealed a strong positive association between oral functional recovery and psychological well-being at six months. WHO-5 scores correlated significantly with mouth opening (r=0.62, p<0.001), masticatory efficiency (r=0.68, p<0.001), and patient-reported oral function scores (r=0.71, p<0.001), as shown in Table 4.

**Table 4:** Correlation Between Oral Functional Parameters and Psychological Well-Being at 6 Months (n=112) Pearson correlation coefficient was used to assess the relationship between oral functional parameters and psychological well-being.

Parameter	Correlation coefficient (r)	p-value
Mouth opening vs WHO-5	0.62	<0.001
Masticatory efficiency vs WHO-5	0.68	<0.001
Speech intelligibility vs WHO-5	0.55	<0.001
Oral function score vs WHO-5	0.71	<0.001

Multivariable linear regression analysis, adjusting for age, sex, smoking status, diabetes mellitus, type of jaw defect, and reconstructive technique, demonstrated that smoking status, diabetes mellitus, masticatory efficiency, and patient-reported oral function were independent predictors of psychological well-being (WHO-5 score) at six months (Table 5). Masticatory efficiency and patient-reported oral function had the strongest positive associations, indicating that better oral functional recovery is linked to improved psychological well-being. The overall model explained 58% of the variance in WHO-5 scores (adjusted R²=0.58).

**Table 5:** Multivariable Linear Regression Analysis for Predictors of WHO-5 Score at Six Months (n=112) β=Unstandardized regression coefficient; 95% CI=95% confidence interval; Reference categories: Female sex, Non-smoker, No diabetes mellitus, Mandibular defect, Local flap reconstruction. Model fit: R²=0.61, Adjusted R²=0.58.

Variable	β coefficient	95% CI	Standard Error	p-value
Age (years)	−0.08	−0.18, 0.02	0.05	0.112
Male sex (ref: Female)	1.94	−0.46, 4.34	1.21	0.109
Smoking status (ref: Non-smoker)	−3.12	−5.62, −0.62	1.38	0.024
Diabetes mellitus (ref: No)	−4.27	−7.90, −0.64	1.69	0.013
Masticatory efficiency	2.86	2.05, 3.67	0.41	<0.001
Oral function score	0.74	0.56, 0.92	0.09	<0.001

## Discussion

The demographic profile of our cohort, characterized by a relatively young mean age and marked male predominance, is consistent with previously published studies on traumatic jaw loss, where young adult males are disproportionately affected due to higher exposure to road traffic accidents and interpersonal violence [17]. The predominance of road traffic accidents as the leading mechanism of injury in our study mirrors reports from South Asian and other low- and middle-income settings, where inadequate road safety measures contribute substantially to maxillofacial trauma burden [1,18]. The higher frequency of isolated mandibular defects compared to maxillary or combined defects also aligns with earlier epidemiological data, given the anatomical prominence and biomechanical vulnerability of the mandible during high-impact trauma [19].

At baseline, patients demonstrated markedly poor psychological well-being, as reflected by low WHO-5 scores, alongside substantial oral functional impairment. These findings are in agreement with prior studies reporting high levels of psychological distress, social withdrawal, and diminished quality of life following traumatic facial disfigurement and loss of oral function [20]. Impaired mastication, restricted mouth opening, and compromised speech collectively affect basic daily activities and social interaction, which are known contributors to depression and anxiety in this patient population [21]. The low preoperative scores in our cohort underscore the profound biopsychosocial impact of traumatic jaw loss prior to definitive reconstruction.

Following reconstruction, we observed significant and progressive improvements in psychological well-being and all oral functional parameters at both three and six months. The magnitude and consistency of improvement across time points support the effectiveness of combined reconstructive and dental rehabilitation approaches. Similar longitudinal improvements in quality of life and mental health outcomes have been reported after mandibular and maxillary reconstruction, particularly when dental rehabilitation is incorporated as part of the reconstructive plan [22]. Our findings extend this evidence by demonstrating that psychological recovery continues beyond the early postoperative period, paralleling functional gains as patients adapt to restored oral competence and facial form.

Improvement in maximal interincisal opening observed in our study is comparable to that reported by previous authors following free flap and regional flap reconstruction, where gradual resolution of fibrosis, improved neuromuscular coordination, and structured postoperative rehabilitation contribute to enhanced jaw mobility [23]. Likewise, the significant gains in masticatory efficiency and speech intelligibility are consistent with earlier reports highlighting the role of stable occlusion, prosthetic rehabilitation, and soft tissue reconstruction in restoring oral performance [1]. Notably, patient-reported oral function scores showed substantial improvement, reinforcing the importance of subjective functional perception alongside objective measurements.

One of the most important findings of this study is the strong positive correlation between oral functional recovery and psychological well-being at six months. The observed associations between WHO-5 scores and mouth opening, masticatory efficiency, speech intelligibility, and patient-reported oral function are in line with previous studies demonstrating that functional restoration is a key determinant of postoperative mental health and social reintegration [18,19]. Patients who regain the ability to eat, speak, and interact confidently are more likely to experience improved self-esteem and emotional well-being, highlighting the holistic benefits of comprehensive reconstruction.

Multivariable regression analysis further demonstrated that masticatory efficiency and patient-reported oral function were independent predictors of psychological well-being, even after adjusting for demographic and clinical confounders. These findings are supported by earlier literature suggesting that the ability to chew effectively and perceive satisfactory oral function has a greater impact on quality of life than anatomical reconstruction alone [11]. The negative association of smoking and diabetes mellitus with psychological outcomes observed in our study has also been reported previously and may be attributed to delayed healing, increased complication rates, and persistent functional limitations in these subgroups [22]. Interestingly, age and sex were not significant predictors, suggesting that functional recovery exerts a stronger influence on psychological outcomes than demographic factors.

Overall, this study reinforces the concept that successful rehabilitation after traumatic jaw loss should be evaluated not only in terms of surgical survival or anatomical reconstruction but also through patient-centered functional and psychological outcomes. By demonstrating the close linkage between oral functional recovery and mental well-being, our findings support an integrated, multidisciplinary approach that prioritizes early dental rehabilitation, structured functional therapy, and psychosocial support. Future studies with longer follow-up and comparative reconstruction strategies may further refine predictors of optimal psychological recovery and guide personalized rehabilitation pathways. This study was conducted at a single center, which may limit the generalizability of the results. The follow-up period was limited to six months, precluding assessment of long-term functional and psychological outcomes. Additionally, although validated tools were used, some measures relied on patient-reported outcomes, which are subject to response bias.

## Conclusions

This prospective study demonstrates that combined dental and facial reconstruction following traumatic jaw loss results in significant and sustained improvements in both oral functional recovery and psychological well-being. Patients initially presented with marked impairment in mastication, speech, mouth opening, and poor psychological health, reflecting the profound biopsychosocial impact of traumatic jaw defects. Post-reconstruction, substantial gains were observed across all functional parameters, accompanied by a parallel improvement in psychological well-being over time. The strong associations between oral functional outcomes and psychological well-being highlight that functional restoration, particularly masticatory efficiency and patient-perceived oral function, is a key determinant of mental and emotional recovery. These findings emphasize that successful reconstruction should not be defined solely by anatomical repair but by meaningful improvements in daily function and psychosocial health. An integrated, patient-centered approach that combines surgical reconstruction, dental rehabilitation, and routine psychological assessment is essential to optimize overall recovery and quality of life in patients with traumatic jaw loss.
